# Smart DNA Fabrication Using Sound Waves: Applying Acoustic Dispensing Technologies to Synthetic Biology

**DOI:** 10.1177/2211068215593754

**Published:** 2016-02

**Authors:** Paulina Kanigowska, Yue Shen, Yijing Zheng, Susan Rosser, Yizhi Cai

**Affiliations:** 1 School of Biological Sciences, University of Edinburgh, The King’s Buildings, Edinburgh, UK; 2 BGI-Shenzhen, Shenzhen, China

**Keywords:** synthetic biology, DNA assembly, acoustic dispensing

## Abstract

Acoustic droplet ejection (ADE) technology uses focused acoustic energy to transfer nanoliter-scale liquid droplets with high precision and accuracy. This noncontact, tipless, low-volume dispensing technology minimizes the possibility of cross-contamination and potentially reduces the costs of reagents and consumables. To date, acoustic dispensers have mainly been used in screening libraries of compounds. In this paper, we describe the first application of this powerful technology to the rapidly developing field of synthetic biology, for DNA synthesis and assembly at the nanoliter scale using a Labcyte Echo 550 acoustic dispenser. We were able to successfully downscale PCRs and the popular one-pot DNA assembly methods, Golden Gate and Gibson assemblies, from the microliter to the nanoliter scale with high assembly efficiency, which effectively cut the reagent cost by 20- to 100-fold. We envision that acoustic dispensing will become an instrumental technology in synthetic biology, in particular in the era of DNA foundries.

## Introduction

Synthetic biology is a nascent interdisciplinary research field that leverages rational design approaches based on engineering principles.^1,2^ Synthetic biology distinguishes itself from traditional genetic engineering in several ways: (1) synthetic biology takes advantage of de novo DNA synthesis technologies, rather than relying on the existing natural templates; (2) synthetic biologists use standardized genetic parts not only to facilitate the assembly of novel sequences, but also to more predictably construct the biological system based on the characterization of individual parts^3^; and (3) similar to other engineering disciplines, computer-assisted designers (CADs) and mathematical modeling are instrumental in synthetic biology to effectively help synthetic biologists navigate the design space.^4^

Although synthetic biology is still in an early stage, several breakthroughs in the past decade have already demonstrated its great potential for society; for instance, Keasling’s group used a synthetic biology approach to engineer the baker’s yeast *Saccharomyces cerevisiae* to produce artemisinin, an important antimalarial drug.^5^ Lu and Collins engineered bacteriophage for an antibiotic therapy^6^ and, more recently, also developed a paper-based cell-free methodology to rapidly detect Ebola viruses.^7^

The enabling technology for synthetic biology is the development of a suite of advanced DNA synthesis and assembly methods, such as Golden Gate assembly,^8^ Gibson assembly,^9^ circular polymerase extension cloning (CPEC),^10^ transformation-assisted recombination (TAR) cloning,^11^ and PaperClip assembly^12^ (for a comprehensive review on DNA assembly methods, refer to Ellis et al.^13^). Collectively, these technologies open up the possibility to redesign and resynthesize DNA at the genome scale. Poliovirus cDNA was synthesized without a natural template in 2002 by Cello et al.^14^ Itaya’s group pioneered the combination of two genomes in one cell in vivo.^15^ The J. Craig Venter Institute chemically resynthesized a bacterial genome^16^ and developed the genome transplantation technology to reboot the cell with the synthetic bacterial genome.^17^ Together with several other groups across the world, our group is part of the international synthetic yeast consortium (www.syntheticyeast.org), which aims to redesign and resynthesize the world’s first eukaryotic genome. We recently reported the completion of the first synthetic yeast chromosome arm^18^ and the first fully synthetic eukaryotic chromosome.^19^ As a safety measurement and responsible innovation in synthetic biology, efficient biocontainment technologies have been developed to restrict the viability of engineered microbes to prevent the dual use of synthetic biology technologies.^20^

Traditional liquid handling technology has enabled increased throughput of many Life Sciences (Lowell, MA) protocols and assays by (1) increasing operational speeds, (2) reducing working volumes (down to a microliter range), and (3) reducing the need for a generally error-prone human handling, and ultimately contributed to substantial workflow cost savings. Despite an already big “leap forward,” the demand for further protocol miniaturization continues to increase, in particular in ultra-high-throughput screening (uHTS).^21^ Traditional tips/nozzles-based robotic platforms struggle to precisely dispense liquid droplets below the microliter threshold. Pin tools can be used to transfer nanoliter to microliter liquid from source plates to destination plates; however, because they are contact based, the pin tools usually require washing and drying between transfers to avoid cross-contamination. Also, the delivery volume of pin tools is difficult to control, as it is due to a combination of many factors, such as the shape of the pin, the diameter of the pin, the coating of the pin, and the speed of dipping and removing of the pin. Finally, pin tools are usually made in 96, 384, and 1536 formats, which limits their flexibility of usage, e.g., in setting up different reaction volumes in the same plate. Another technology allowing reaction miniaturization is the microfluidic chip technology.^22^ Kong and others have successfully used microfluidic chips to synthesize DNA sequences up to 1 kb,^23^ and Tewhey et al. used microfluidic chips to run 1.5 million PCRs in parallel.^24^ The main disadvantage of the microfluidic chip approach is that the master molds and the control layer need to be custom designed and fabricated for different reactions; however, the de novo DNA synthesis using microfluidic chips is very complementary with the miniaturized assembly methods described in this paper.

First described in 1927, the acoustic droplet ejection (ADE) phenomenon utilizes acoustic energy to rapidly move low-volume nanoliter to picoliter droplets without any physical contact.^25^ Before it reached the laboratory setting in the 2000s, the drop-on-demand technology was first exploited in a number of other fields, including the ink-jet printing industry. Today, Labcyte, Inc. (Sunnyvale, CA) is pioneering the acoustic dispensing technology for Life Sciences, with its Echo series robotic platforms being able to transfer multiple 2.5 or 25 nL droplets from the 384- and 1536-well sources to the various (inverted) destination plates. Unlike traditional robotic liquid transfer methods, laboratory acoustic dispensing has been shown to be highly precise at the nanoliter volume range (as demonstrated by its low coefficients of variation), therefore enabling the desired further miniaturization of current protocols and assays. The acoustic dispenser is flexible enough to set up any-to-any configurations between the source plate and the destination plate, and the reaction volumes can vary from well to well in the same reaction plate.

Here, for the first time, we report yet another exciting acoustic dispensing application: nanoliter-scale DNA assembly. The majority of assembly expenses are enzymes, including DNA polymerases, Therefore, downscaling the reaction volume from the microliter to the nanoliter scale while maintaining high assembly efficiency, will make DNA synthesis and assembly more accessible to synthetic biologists.

## Materials and Methods

### Echo PCR

**Figure 1.**
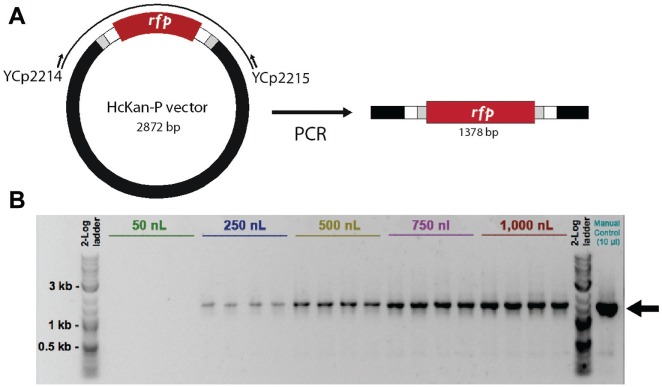
PCR setup by Echo. **(A**) A pair of primers was designed to amplify a fragment of 1.3 kb, and PCRs of various volumes were set up by the Echo machine. (**B**) Gel electrophoresis confirms that PCR can work at the 250 nL scale.

Conventional endpoint PCR is instrumental in making synthetic DNA. To test the minimal volume of regular PCR using Echo, we set up PCRs of various volumes. The plasmid HcKan_P vector (120 ng/µl) was used as the DNA template, and a pair of primers YCp2214 and YCp2215 were designed to amplify a targeted DNA fragment of 1378 bp (**Fig. 1A**; all primers used in this paper are listed in **Suppl. Table S1**). The GoTaq Green Master Mix (Promega, Madison, WI) was used in the PCR. Five reaction volumes ranging from 50 to 1000 nL were set up (**Table 1**), and each reaction was performed in four replicates. All PCRs were set up using the following cycling conditions: preheat the PCR machine and then put in the PCR plate, 2 min at 95 °C, 32 cycles of 10 s at 95 °C, 30 s at 50 °C and 2 min at 72 °C, 7 min at 72 °C, and hold at 4° C. GoTaq Green Master Mix (35 µL) and double-distilled water (ddH_2_O; 30 µL) were added separately to source plate 1, which was an Echo 384-well polypropylene plate (Labcyte). YCp2214 and YCp2215 (10 µL each) and template DNA (10 µL) were added separately to source plate 2, which was an Echo 384-well low-dead-volume plate (Labcyte). The destination plate used in this paper was MicroAmp EnduraPlate (Life Technologies, Carlsbad, CA).

**Table 1.** PCR Setup.

Reagent/nl	Echo	Echo	Echo	Echo	Echo	Manual
Primer YCp2214	2.5	12.5	25.0	37.5	50.0	500.0
Primer YCp2215	2.5	12.5	25.0	37.5	50.0	500.0
Template DNA	5.0	25.0	50.0	75.0	100.0	1000.0
ddH_2_O	15.0	75.0	150.0	225.0	300.0	3000.0
GoTaq Green Master Mix	25.0	125.0	250.0	375.0	500.0	5000.0
**Total**	50 nL	250 nL	500 nL	750 nL	1000 nL	10,000 nL

### Gibson DNA Assembly

First described in 2009, the Gibson DNA assembly method^9^ belongs to a group of overlap-directed DNA assembly techniques such as CPEC,^10^ SLiCE,^26^ and SLIC^27^ assemblies. The Gibson assembly method is one of the most used in synthetic biology, and it can assemble DNA sequences up to small genome sizes from overlapping DNA fragments in an isothermal one-pot reaction. The advantage of Gibson assembly is that it is sequence independent and generates scarless final assembled DNA products. Typically, the Gibson assembly requires about a 40 bp homologous region between two adjacent DNA fragments, and these homologous regions are usually added to the fragments by a high-fidelity PCR. Briefly, the assembly reaction takes place in a cocktail of enzymes (termed Gibson master mix) at 50 °C for 60 min: (1) First, T5 exonulease chews back the DNA in a 5′ to 3′ direction from the homologous terminal ends to reveal reverse complementary single-stranded sequences between two adjacent fragments. (2) While the 5′ to 3′ DNA digestion proceeds, a high-fidelity DNA polymerase fills in the single-stranded DNA region. (3) Finally, Taq DNA ligase seals the nicked DNA strands, which yields the final assembled product.

#### Gibson Reaction Setup by Echo

Two pairs of primers (YCp2391 and YCp2392 for fragment 1, YCp2393 and YCp2394 for fragment 2) were designed to amplify two fragments with 40 bp end homology from a red fluorescent protein (RFP)–containing plasmid pPC025, thus allowing subsequent Gibson reassembly of the plasmid. The two homologous junctions were placed within the ampicillin resistance gene and the RFP open reading frame (ORF) to reduce the overall false positive rate and to allow phenotypic screening for successful assembly isolates, respectively. In contrast to Golden Gate assembly (see below), here RFP serves as a positive screen for correct assemblies. PCR products were gel purified using the QIAquick gel extraction kit (Qiagen, Valencia, CA). The standard 15 µL Gibson assembly master mix was prepared as described in the original Gibson assembly paper.^9^ Gibson master mix (40 µL) was added to source plate 1, which is an Echo 384 polypropylene plate (Labcyte). Each DNA fragment (10 µL) was added to source plate 2, which is an Echo 384 low-dead-volume plate (Labcyte). One-pot Gibson assembly was incubated at 50 °C for 60 min in a preheated PCR thermal cycler (**Table 2**).

**Table 2.** Gibson Assembly Reactions.

Reagent/nl	Echo	Echo	Echo	Echo	Manual
Gibson master mix	37.5	187.5	375.0	750.0	15,000.0
Fragment 1 (113.8 ng/µl)	5.0	20.0	40.0	80.0	2,500.0
Fragment 2 (86.8 ng/µl)	7.5	42.5	85.0	170.0	2,500.0
**Total**	50 nL	250 nL	500 nL	1000 nL	20,000 nL

### Golden Gate Assembly

The Golden Gate DNA assembly method utilizes a combination of a TypeIIS restriction enzyme and a ligase to assemble the DNA fragments.^8^ TypeIIS enzymes (e.g., BsaI and BsmBI enzymes) are endonucleases that cut outside their recognition sites, creating 4 bp DNA overhangs. By carefully designing the 4 bp overhangs, one can use the Golden Gate reaction to directionally assemble DNA fragments. The Golden Gate DNA assembly reaction starts with a given TypeIIS endonuclease DNA digestion, leaving behind staggered cuts in the backbone and the fragment DNA. The design-imposed DNA complementarity allows annealing of the resulting “sticky ends,” creating the desired plasmid construct. In the final reaction step, the T4 DNA ligase repairs the nicks to complete the DNA construction phase.

#### Golden Gate Reaction Setup by Echo

**Figure 2.**
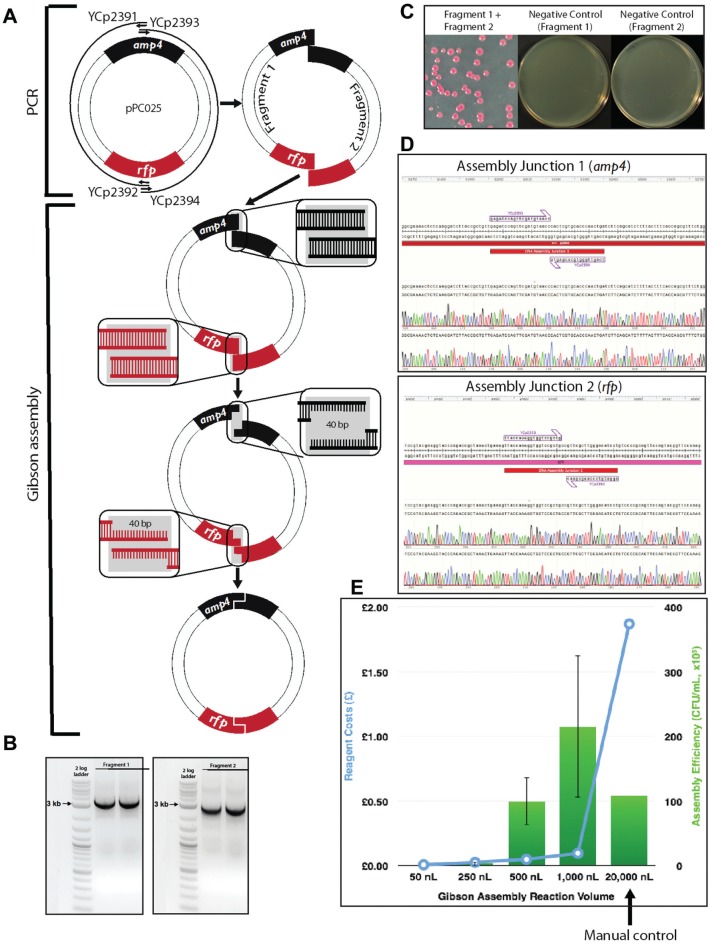
Gibson assembly reaction setup by Echo. (**A**) The pPC025 plasmid was split into two overlapping fragments at the middle of the ampicillin resistance gene and the RFP ORF. Two fragments were generated with 40 bp overlap at both ends and then assembled by the Gibson assembly reaction. (**B**) Gel electrophoresis confirming the successful PCR amplification of both fragments. (**C**) Successful Gibson assembly product gives rise to red bacterial colonies. The assembly efficiency was so high that no background colonies (white) were observed. Negative control reactions, which had only one fragment in the reactions, yielded no colonies. (**D**) Sequencing verification of both assembly junctions shows 100% assembly accuracy. (**E**) Cost-effectiveness and assembly efficiency comparison of different reaction volumes for Gibson assembly.

The HcKan_P plasmid (2.8 kb, diluted to 10 ng/µl) was used as the acceptor vector. This plasmid carries a KanR selectable marker, along with a RFP cassette flanked by a pair of outward-facing BsaI sites. We amplified the promoter *pMBP1* (500 bp) directly from yeast BY4741 (MATa, *leu2∆0 met15∆0 ura3∆0 his3∆1*) genomic DNA with primers YCp2395 and YCp2396 and added a pair of inward-facing BsaI sites to flank the promoter part (**Fig. 2A**). The PCR product was purified using a PureLink PCR purification kit (Life Technologies) and diluted to 20 ng/µl. The 4 bp overhangs were designed in such a way that the promoter can be efficiently assembled into the acceptor vector. Bacteria carrying the residual RFP plasmid will give a bright red pigment, which would facilitate the visual identification of correct assembled clones (white colonies; see **Fig. 2C**).

The Golden Gate master mix was made of 35 µL T4 ligase (2000 U/µl, New England Biolabs, NEB), 35 µL BsaI-HF (NEB), 52.5 µL 10× T4 buffer (NEB), and 25 µL 200× BSA (NEB). Golden Gate assembly reactions were set up using the following cycling conditions: 15 cycles of 5 min at 37 °C and 10 min at 16 °C, 5 min at 50 °C, 10 min at 80 °C, and hold at 4 °C. Five reaction volumes arranging from 50 to 1000 nL were set up (**Table 3**), and each reaction was performed in triplicate. A manual positive control reaction of 7.5 µL was also set up to confirm the fidelity of the reagents. Golden Gate master mix (30 µL) was added to source plate 1, which is an Echo 384 polypropylene plate (Labcyte). *pMBP1* PCR product (10 µL) and HcKan_P vector (10 µL) were added to source plate 2, which is an Echo 384 low-dead-volume plate (Labcyte).

**Table 3.** Golden Gate Assembly Reactions.

Reagent/nl	Echo	Echo	Echo	Echo	Manual
Golden Gate master mix	17.5	82.5	167.5	332.5	2500
pMBP1 (20 ng/µl)	30.0	150.0	300.0	600.0	4500
HcKan_P (10 ng/µl)	2.5	17.5	32.5	67.5	500
**Total**	50 nL	250 nL	500 nL	1000 nL	7500 nL

### Bacterial Transformation

As the assembly reactions set up by Echo were at the nanoliter scale, it is difficult to take out the assembled DNA using pipets and transform them into bacterial competent cells. Instead, bacterial competent cells were added to each well containing an assembled product. Competent *Escherichia coli* (20 µL; MAX Efficiency DH5α, Life Technologies) was added to each well of the reaction plate. The PCR plate was incubated on ice for 20 min and then placed in a heat block at 42 °C for 45 s. The plate was placed back on ice to incubate for 5 min, before adding 200 µL of room temperate *super Optimal Catabolite repression (SOC)* medium to each well. The plate was incubated at 37 °C with shaking at 200 rpm for 1 h. A multichannel pipet was used to slowly drip 40 µL of each transformation mixture onto an omnitray containing selective solid agar medium (LB—Kan). Alternatively, 100 µL of transformation mixture was plated on individual petri dishes with selective solid agar medium (Golden Gate assembly, LB—Kan; Gibson assembly, LB—Amp). Plates were incubated overnight at 37 °C until single colonies appeared.

### Gel Electrophoresis

Gel electrophoresis was performed to analyze the PCR products (120 V, 30 min; 1% w/v agarose in Tris-acetate-EDTA (TAE) buffer with 1× SYBR Safe DNA stain). Each PCR product was first diluted with ddH_2_O to a final volume of 5 µL when the PCR volume was smaller than 5 µL.

### Sanger Sequencing

A BigDye Terminator v3.1 Cycle Sequencing Kit (Life Technologies) was used to verify the DNA assembly clones according to the manufacturer’s instructions, and the Sanger sequencing reactions were carried out by Edinburgh Genomics.

## Results and Discussion

We used the Echo machine to set up PCRs in total volumes ranging from 50 nL to 1 µL (**Fig. 1** and **Table 1**). Starting from 250 nL, a band of the correct size could be detected in the gel electrophoresis. Because we diluted the PCR product to 5 µL in order to run the gel electrophoresis, it is possible that PCRs at 50 nL scale were successful, but the gel electrophoresis was not sensitive enough to detect the signal. Alternatively, it would be possible to use the Caliper Labchip GX instrument that can detect DNA concentrations as low as 5 ng/µL. Downsizing the PCR from 50 µL or higher to 250 nL already effectively cuts the reagent cost by 200-fold. Miniaturized PCR is ideal for diagnostic purposes such as fast genotyping and colony-screening PCR, but it is less suitable for applications requiring use of the PCR product for downstream procedures, such as cloning, because the yield of double-stranded DNA may not be sufficient.

Gibson assembly worked extremely well in this experiment. Correct assembly was observed from as low as the 250 nL reaction volumes, and at 500 and 1000 nL the assembly efficiencies are comparable with or better than the manual control of the 20 µL reaction, but with a significant standard deviation. This allows us to cut the reagent cost by 20-fold or more. Even more encouraging, we observed no background (**Fig. 2C**) and 100% correct assembly through Sanger sequencing across the assembly junctions (six red colonies were sequenced; **Fig. 2D**), and this will be highly beneficial for future automation plans, as it will greatly reduce the colony screening effort.

**Figure 3.**
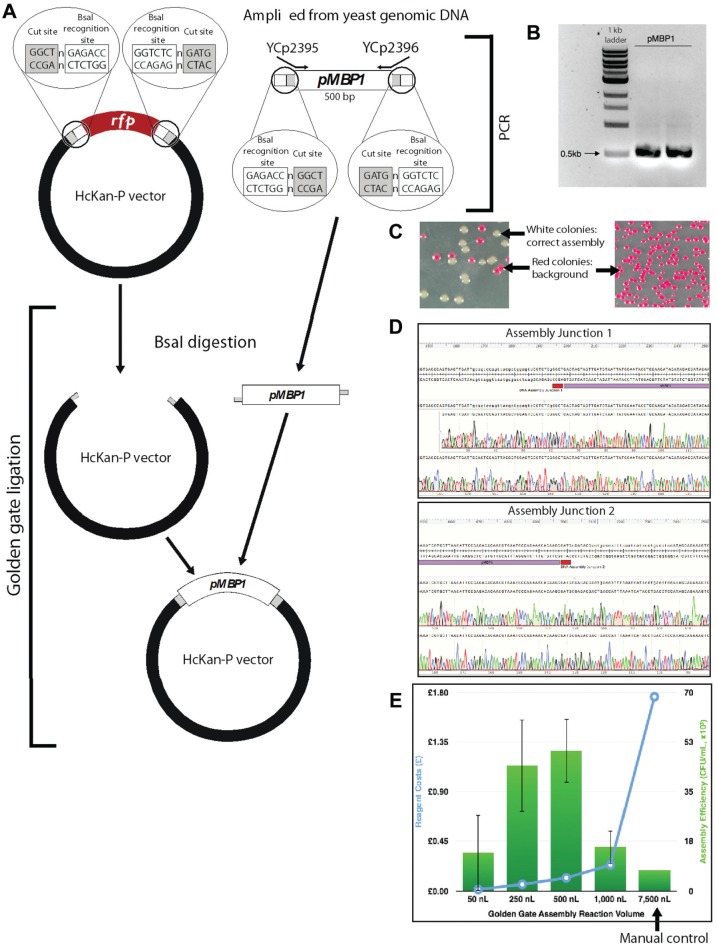
Golden Gate assembly setup by Echo. (**A**) A promoter pMBP1 was amplified from the yeast genome to add appropriate Golden Gate sequences (BsaI recognition sites + 4 bp overhangs). The acceptor vector HcKan_P plasmid carries a RFP cassette, which is flanked by corresponding Golden Gate sequences to uptake the pMBP1 part in the Golden Gate reaction. (**B**) Gel electrophoresis indicates successful amplification of pMBP1. (**C**) Left: Successful assembled DNA gives rise to white colonies, while the residual acceptor vector yields red colonies. Right: Negative control, which contained only the acceptor vector in the Golden Gate reaction, yielded only red colonies. (**D**) Sequencing verification of both assembly junctions shows 100% assembly accuracy. (**E**) Cost-effectiveness and assembly efficiency comparison of different reaction volumes for Golden Gate assembly.

With Golden Gate assembly, we successfully assembled DNA at a 50 nL reaction volume (typically 15 µL reactions when performed manually), and at the 250 and 500 nL scales the assembly efficiencies are higher than those of the manual control. This leads to at least a 30-fold reduction in reagent use when performing Golden Gate reactions using Echo. We did observe vector background in the assembly (red colonies, as shown in **Fig. 3C**). There are several ways we can overcome this problem. First, instead of using RFP for screening, we can use the toxic ccdB gene, which cannot give rise to background colonies in a nonpermissive transformation host. Second, we can add a higher concentration of the BsaI enzyme in the Golden Gate master mix to further digest the residual acceptor vector. Finally, we may be able to reduce the background by extending the BsaI digestion step in the incubation.

With further optimization, it should be possible to downsize the reaction volume even further. For instance, in this paper, individual components were shot off to the destination well one by one (in the case of 50 nL PCRs, only 1 droplet of primer was shot), and it is possible that some components were not sent into the reaction pool due to slight misalignment of the acoustic dispenser, meaning the reactants simply didn’t mix. In this case, it would be of advantage to premix as many components as possible and then shoot more droplets altogether. We also suggest dispensing the master mix using a bulk dispenser or liquid handler, so that the destination well has a larger liquid surface to uptake the incoming droplet and minimize chances for the droplets hitting the well wall. It is always a good practice to centrifuge the PCR plate when appropriate before putting it into the PCR thermal cycler to start the reaction. To prevent the nanoliter droplet from evaporating before the chemical reaction starts, we always preheat the PCR machine before putting in the reaction plate. Finally, it is more economical to use low-dead-volume plates as the source plate for expensive reagents such as enzymes and polymerases.

In the world of laboratory automation, efficiency and robustness are as important as cost saving. With this in mind, we overlaid the number of correct assemblies (efficiency) with standard deviation (robustness) in the same plot with the cost of reactions for the Gibson assembly (**Fig. 2E**) and the Golden Gate assembly (**Fig. 3E**). The intersections of the two curves indicate the “sweet spots” for choosing desired reaction volumes, which are of high efficiency, low standard deviation, and relatively low cost. It should be noted that our cost calculation did not take into account the dead volume of reagents, and logically it can be assumed that the dead-volume cost per reaction would decrease as more reactions are set up by Echo in one experiment. Whenever possible, low-dead-volume plates should be used for expensive reagents to save cost. Conversely, we did not include the tip cost in the manual control experiments, which increase substantially when the number of reactions is scaled up. Continuously monitoring DNA assembly efficiency along with the assembly cost is critical to successful operation of a large DNA synthesis and assembly automation facility, such as the UK DNA foundries.

The acoustic dispensing has great potential in automating other molecular biology operations. We also used the Echo to purify single colonies from bacterial and yeast cultures, which is traditionally challenging to automate. As Echo is capable of dispensing nanoliter droplets with high precision, it is also ideal for generating high-density assembly libraries through combinatorial assembly methods. In conclusion, the work described here is the first report on use of the acoustic dispenser in the area of synthetic biology, and we envision that this technology will be instrumental in lab automation, in particular in the era of DNA foundries.

## Supplementary Material

Supplementary material
