# Supplementary material for: Smart DNA Fabrication Using Sound Waves: Applying Acoustic Dispensing Technologies to Synthetic Biology
Source: J Lab Autom. 2016 Feb;21(1):49–56. doi: 10.1177/2211068215593754 (PMC4814025; doi:10.1177/2211068215593754)
Supplement: Supplementary material [file DS_10.1177_2211068215593754.pdf]

Supplemental Data

Table S1: DNA Assembly Reagent Costs*

Reagent Name	Supplier	Product Code	Units	Pack Size	Pack Price (£)	Price/Unit (£)	Additional Information
UltraPure 1 M Tris-HCI Buffer, pH 7.5	Life Technologies	15567027	uL	1,000,000	47.38	0.00004738	-
Magnesium chloride solution, for molecular biology, 1.00 M±0.01 M	Sigma-Aldrich	M1028-100ML	uL	100,000	50.1	0.000501	-
Deoxynucleotide Mix, PCR-Grade, 400 uL	Agilent Technologies	200415	uL	400	125.1	0.31275	100 uL/dNTP tube x 4
DL-Dithiothreitol, for molecular biology, =98% (TLC), =99% (titration)	Sigma-Aldrich	D9779-5G	G	5	86.77	17.354	MW=154.25 g/mol
Poly(ethylene glycol), BioUltra, 8,000	Sigma-Aldrich	89510-250G-F	G	250	17.9	0.0716	-
ß-Nicotinamide adenine dinucleotide hydrate, =99%	Sigma-Aldrich	N1511-1G	g	1	85.1	85.1	MW=664.43 g/mol
T5 Exonuclease	NEB	M0363L	U	5,000	163.2	0.03264	10 U/uL
Phusion High-Fidelity DNA Polymerase	NEB	M0530L	U	500	250.4	0.5008	2 U/uL
Thermus Aquaticus (Taq) DNA Ligase	NEB	M0208L	U	10,000	195.2	0.01952	40 U/uL
T4 DNA Ligase	NEB	M0202M	U	100,000	166.4	0.001664	c=2,000 U/uL
BsaI-HF	NEB	R3535L	U	5,000	169.6	0.03392	c=5 U/uL
BSA, Molecular Biology Grade	NEB	B9000S	mg	12	15.2	1.266666667	c=0.02 mg/uL

*based on our quoted prices

Table S2: Gibson DNA Assembly Costs

Reagent Name	Reaction Volume (nL)	Cost (£)
**UltraPure 1 M Tris-HCI Buffer, pH 7.5**	50	2.369×10^{-7}
	250	1.1845×10^{-6}
	500	0.000002369
	1,000	0.000004738
	20,000 (Manual Control)	0.00009476
**Magnesium chloride solution, for molecular biology, 1.00 M±0.01 M**	50	2.505×10^{-7}
	250	1.2525×10^{-6}
	500	0.000002505
	1,000	0.00000501
	20,000 (Manual Control)	0.0001002
**Deoxynucleotide Mix, PCR-Grade, 400 uL**	50	0.0001251
	250	0.0006255
	500	0.001251
	1,000	0.002502
	20,000 (Manual Control)	0.05004
**DL-Dithiothreitol, for molecular biology, =98% (TLC), =99% (titration)**	50	6.68997×10^{6}
	250	3.34498×10^{-5}
	500	6.68997×10^{-5}
	1,000	0.000133799
	20,000 (Manual Control)	0.002675987
**Poly(ethylene glycol), BioUltra, 8,000**	50	0.000000179
	250	0.000000895
	500	0.00000179
	1,000	0.00000358
	20,000 (Manual Control)	0.0000716
**ß-Nicotinamide adenine dinucleotide hydrate, =99%**	50	2.82715×10^{-6}
	250	1.41357×10^{-5}
	500	2.82715×10^{-5}
	1,000	5.6543×10^{-5}
	20,000 (Manual Control)	0.00113086
**T5 Exonuclease**	50	0.000006528
	250	0.00003264
	500	0.00006528
	1,000	0.00013056
	20,000 (Manual Control)	0.0026112
**Phusion High-Fidelity DNA Polymerase**	50	0.000626
	250	0.00313
	500	0.00626
	1,000	0.01252
	20,000 (Manual Control)	0.2504
**Thermus Aquaticus (Taq) DNA Ligase**	50	0.003904
	250	0.01952
	500	0.03904
	1,000	0.07808
	20,000 (Manual Control)	1.5616
**TOTAL Cost (£)**	**50**	0.004671812
	**250**	0.023359058
	**500**	0.046718115
	**1,000**	0.09343623
	**20,000 (Manual Control)**	1.868724607

Table S3: Golden Gate DNA Assembly Costs

Reagent Name	Reaction Volume (nL)	Cost (£)
**T4 DNA Ligase**	50	0.011093333
	250	0.055466667
	500	0.110933333
	1,000	0.221866667
	7,500 (Manual Control)	1.664
**BsaI-HF**	50	0.000565333
	250	0.002826667
	500	0.005653333
	1,000	0.011306667
	7,500 (Manual Control)	0.0848
**BSA, Molecular Biology Grade**	50	6.02933×10^{-5}
	250	0.000301467
	500	0.000602933
	1,000	0.001205867
	7,500 (Manual Control)	0.009044
TOTAL Cost (£)	**50**	**0.01171896**
	**250**	**0.0585948**
	**500**	**0.1171896**
	**1,000**	**0.2343792**
	**7,500 (Manual Control)**	**1.757844**

Table S4: Echo 550 (Labcyte Inc.) Fluid Transfer Specifications

**Manufacturer's Specifications**	
Drop Transfer Volume:	2.5 nL
Volume Transfer Range	2.5 to 10,000 nL (higher volume transfers with an extended transfer time)
Transfer Accuracy	<10% deviation from target volume
Transfer Precision	<8% CV (coefficient of variation)

Table S5: Acoustic Dispensing Labware Used

Plate Type	Plate Name	Supplier	Catalogue #	Format	Working Range	Sterilization	Comments
Source Plate	Echo® Qualified 384-Well Polyproplene Source Microplate (384PP)	Labcyte Inc.	P-05525	384-well	Buffer/protein Working Range: 20 – 50 µL	Non-sterile	Used for the dispensing of master mixes
Source Plate	Echo® Qualified 384-Well COC Source Microplate, Low Dead Volume (384LDV)	Labcyte Inc.	LP-0200	384-well	Buffer Working Range: 3 – 12 µL	Non-sterile	Used for the dispensing of DNA solutions
Destination Plate	MicroAmp® EnduraPlate™ Optical 96-Well Multicolor Reaction Plates with Barcode	Life Technologies	4483355	96-well	N/A	Non-sterile	-

Table S6: Echo 550 Fluid Calibrations Used

Calibration Type	Plate Type	Manufacturer's Description
384LDV_AQ_B	384LDV	Simple buffers: PBS, cell culture media without protein, cDNA, primers/probes
384PP_AQ_SP2	384PP	Buffers, reagents containing surfactant (e.g. PCR mastermix, lysis buffers, reagents with Triton X- 100, Tween-20, SDS, NP-40,etc.), with or without serum/plasma.
384PP_AQ_CP	384PP	Reagents without surfactants. MPD/PEGS/Osmotic solutions. Solutions reported in MRayl.

Table S7: Primers Used

ID	Usage	Orientation	Tm (°C) *	Sequence (5' – 3')	Concentration (uM)
YCp2214	PCR/Golden Gate Colony Verification	Forward	49	gatcctttgattttctaccg	10
YCp2215		Reverse	48	ctcgataactcaaaaaatacg	10
YCp2391	Gibson Assembly Fragment PCR	Forward	53	gagatccagttcgatgtaacc	10
YCp2392		Reverse	55	aggatgtcccaagcgaac	10
YCp2393		Forward	58	ttaccaaaggtggtccgctg	10
YCp2394		Reverse	58	tcagttgggtgcacgagtg	10
YCp2395	Golden Gate Assembly gDNA PCR	Forward	60	agcgtgggtctcgggctactagtagttgatctaattatggaatacc	10
YCp2396		Reverse	63	gtgctgggtctcacatcgcttgtgtttctgggatttacgttgtgtc	10
YCp2568	Gibson Assembly Colony Verification	Forward	54	cttactgtcatgccatccg	10
YCp2569		Reverse	52	cgctcatgagacaataacc	10
YCp2570		Forward	53	gcttctagagcaatacgca	10
YCp2571		Reverse	54	gaagttggtaccacgcag	10

*as indicated by the SnapGene software
